# Three years of experience with a workshop for medical specialty examiners in South Baden: A project report and initial evaluation results

**DOI:** 10.3205/zma001132

**Published:** 2017-11-15

**Authors:** Irmgard Streitlein-Böhme, Wilhelm Niebling, Götz Fabry, Klaus Böhme

**Affiliations:** 1 Universität Freiburg, Lehrbereich Allgemeinmedizin, Freiburg, Germany; 2 Universität Freiburg, Freiburg, Germany; 3 Universität Freiburg, Abt. für Med. Psychologie, Freiburg, Germany

**Keywords:** Medical specialty exam, oral assessments, examiner workshop, post-graduate training

## Abstract

**Introduction: **An oral exam (30-60 minutes) is administered at the end of every post-graduate medical specialty program or is required to attain additional specialized qualifications. In both undergraduate and post-graduate medical education oral exams are not considered to be very objective or reliable. To improve the quality of exams in medical specialties, the Regional Medical Association for South Baden (Bezirksärztekammer Südbaden) decided in 2013 to offer a training program for head examiners and others responsible for administering exams in medical specialties.

**Project Description: **Following a survey of examiners and examinees conducted from January through June, 2013, on the difficulty level of examination questions, satisfaction with the test, and the need for training in administering exams, the first workshop of its kind was designed. Since 2013, six workshops with a total of 93 participants have been held and evaluated.

**Results: **The evaluations (response rate: 86%) showed a high level of acceptance for the concept behind the training. A large number of participants felt the need to define minimum standards for exams, to standardize the required level of difficulty and the assessment criteria in each subject, and to give examiners the appropriate tools needed to improve the validity and reliability of the exams.

**Conclusion: **Offering a training program for those responsible for administering medical specialty exams appears to be both meaningful and necessary in order to meet the existing need for increased validity and reliability. In light of the initial experiences with this workshop and the differing percentages of failed exam attempts nationwide, the implementation of examiner training is to be recommended in other regions in Germany. In other European countries examiners conducting medical specialty exams undergo appropriate training before administering their first exam.

## Zusammenfassung

**Einleitung:** Eine 30- bis 60-minütige mündliche Prüfung steht am Ende jeder Facharztweiterbildung bzw. ist für den Erwerb von Gebiets-, Schwerpunkts- und Zusatzbezeichnungen erforderlich. In der medizinischen Aus- und Weiterbildung gelten mündliche Prüfungen als wenig objektiv und reliabel. Um eine Verbesserung der Qualität von Facharztprüfungen zu erreichen, hat sich die Bezirksärztekammer Südbaden 2013 entschieden, eine Qualifizierungsmaßnahme für FacharztprüferInnen und Prüfungsvorsitzende anzubieten.

**Projektbeschreibung: **Nach einer von Januar bis Juni 2013 durchgeführten Befragung der PrüferInnen, Prüfungsvorsitzenden und Prüflinge u.a. zum Niveau der Prüfungsfragen, zur Zufriedenheit mit der abgelaufenen Prüfung sowie zum prüfungsmethodischen Fortbildungsbedarf, erfolgte die Konzeption eines bundesweit ersten Workshop-Angebotes. Seit 2013 wurden 6 Workshops mit insgesamt 93 TeilnehmerInnen durchgeführt und evaluiert.

**Ergebnisse: **Die Evaluationen (Rücklauf: 86%) zeigten eine hohe Akzeptanz des Schulungskonzeptes. Eine große Anzahl von TeilnehmerInnen sahen die Notwendigkeit, Minimalstandards für die Prüfungen festzulegen, das Anforderungsniveau und Bewertungskriterien in den jeweiligen Fächern zu vereinheitlichen und den FacharztprüferInnen entsprechende Instrumente an die Hand zu geben, um Validität und Reliabilität der Prüfungen zu verbessern.

**Schlussfolgerung:** Das Angebot eines Schulungsprogramms für FacharztprüferInnen und Prüfungsvorsitzende erscheint sinnvoll und notwendig, um einem bestehenden Bedarf an mehr Validität und Reliabilität zu entsprechen. Nach den ersten Erfahrungen mit diesem Workshop-Angebot und vor dem Hintergrund der bundesweit sehr unterschiedlichen Nichtbestehensquoten wäre die Durchführung von Qualifizierungsmaßnahmen von FacharztprüferInnen auch in anderen Ärztekammerbezirken zu empfehlen. In verschiedenen europäischen Ländern durchlaufen FacharztprüferInnen bereits vor ihrem ersten Einsatz entsprechende Programme.

## Introduction

In Germany oral assessments have a long tradition not only in undergraduate medical education, but also in post-graduate medical specialization. However, the validity and reliability of oral exams have been viewed as overall low, primarily because they are very susceptible to grading errors and biases. This has been demonstrated by numerous studies [1], [2]. In particular, the tendency described by Wakeford et al. to test only for factual knowledge, not to give separate scores for partial performance and the “minor errors” belong to the most important examples of grading biases and errors that can occur in the context of a medical specialty exam. Wakeford et al. see a solution in the selection and training of examiners. In the training sessions the examiners should measure themselves against the exam criteria [2].

Jefferies et al. also consider it important to explore ways to increase the quality of practical exams, above all, to implement a stronger focus on competency [3].

To achieve a higher degree of objectivity, reliability and thus validity in an oral exam, it is necessary to structure and standardize both the test situation and the test questions [4], as previously called for in 1999 in the recommendations regarding structured oral assessments in the medical specialty exams. These stress the necessity of ensuring identical test conditions for examinees in order to achieve standardization of the exam. It is therefore necessary to define and weight the assessment objectives and content in advance for each particular medical specialty exam. It would be most logical if all of the examiners agreed upon exam content and identified which grading criteria apply to candidates who fail the exam. An improvement in structuring will be evident mainly in a written record of the test questions formulated in advance along with the corresponding expectations regarding the correct answers and a scoring rubric. The exam questions should be case-based and contain aspects relevant to practice (e.g. x-rays, ECGs, sonographic images, etc.).

Currently in Germany there are only few examples of how to improve the medical specialty exam. For instance, in Thuringia a change was made to the exams in 2014 in response to demands made by examiners: topics and sets of questions were defined to better standardize the exam, and exam content was modernized, for example, through digital images in radiology or anchoring practical components in the exam. As a result, the 30-minute timeframe for the exam was extended to 45-90 minutes per examinee [5].

The question arises as to which improvements in assessment quality are possible within the existing situation. Among different European countries (Denmark, Germany, The Netherlands, Poland, and Great Britain) some mandatory training courses for examiners have been established. This was ascertained as part of a pilot survey of an expert panel consisting of examiners from five major EU countries who were responsible for administering the medical specialty exam in general practice. Only in Poland and Germany was this kind of training not offered. In addition, the survey revealed that all of the countries except for Germany carry out longitudinal evaluation of performance over the entire course of post-graduate medical training [6].

That training sessions can completely change grading behavior is shown by an analysis of scoring on the oral/practical M2 state examinations at the end of undergraduate medical education. Schickler et al. identified differences in the scores of examinees who were evaluated by examiners with or without prior training. The highest grade (“very good”) was given much less often by examination committees with members who had attended a workshop addressing the issue of grading [7].

### Initial situation in South Baden

In April 2012 as part of an extraordinary meeting of the Regional Medical Association for South Baden addressing the current state of post-graduate clinical and practical training, the decision was made to define (minimum) standards for medical specialty exams and to initiate dialogue among the examiners [8]. As a consequence, in 2013 the Regional Medical Association offered a workshop for the first time aimed at examiners responsible for administering medical specialty exams. The most urgent goal of the workshop was to effect qualitative improvements in the content and method of the 30- to 60-minute specialty exam administered in South Baden.

The first step involved evaluating the medical specialty exams. From January to June, 2013, the head examiners, examiners and examinees were surveyed immediately after the 102 medical specialty exams administered during this period. The questionnaire with 13 items, plus open-ended questions, was primarily focused on the level of difficulty of the exam questions, the level of satisfaction with the assessment, and the need for extra training on the topic of assessments.

The qualitative analysis of the initial evaluations after the medical specialty exams showed that only 27.3% of the head examiners and 23.4% of the examiners saw a need to train examiners to administer the medical specialty exams [8]. The open-ended responses, however, indicated numerous suggestions for content-based and structural improvements to the previous medical specialty exams. For example, 20.8% of examiners and 36.4% of the head examiners desired a standardization of the exam questions for each subject area and/or determination of grading criteria prior to the exam. A total of 7.8% of examiners felt 30 minutes for a medical specialty exam were too short. The main criticisms were mainly the varying levels of difficulty of the exam questions, the absence of coordination among examiners regarding exam content, and the lack of required subject-specific content and grading criteria.

With only a few exceptions the examinees demonstrated a high level of acceptance for the exams that had taken place. It must be pointed out that the annual failure rate for the medical specialty exams in South Baden is <3% [8].

In view of the high level of satisfaction among the examiners and head examiners with the mode of testing at the time, the question arose whether this group would be accepting of a training measure offered by the Regional Medical Association precisely for examiners. What could the content of such a training program encompass and which improvements could be achieved as a result?

## Project Description

Based on the decision of the representatives and the executive board of the Regional Medical Association for South Baden, supported by international examples [2], [3], [4] and a wide variety of suggestions for improvement made by examiners in the initial survey, a training program comprised of six units (4.5 hours) was developed at the beginning of 2013 and heavily influenced by the workshop offered at the time by the medical schools in Baden-Württemberg for examiners conducting the oral/practical section of the M2 exam (now the M3 exam) [9]. Adaptation for the medical specialty exams is shown in table 1 (Tab. 1), and the most important improvements described above were implemented in the medical specialty exams:

Prior to the workshop, each participant received example materials and was asked to prepare two case vignettes with exam-relevant patient cases from practice, including the grading criteria for correct answers, and to bring other exam materials such as x-ray images, ECG leads, and lung function results to the workshop. The case vignettes were first reviewed internally as part of the workshop to then be used later as exam questions during the subsequent exam simulation.

The workshop has taken place six times since 2013 with a total of 93 participants coming from a wide range of medical disciplines and has been evaluated each time at the end. During this time period it has been slightly modified and adjusted for the particular number of attendees and composition of medical disciplines.

A participant evaluation was administered at the end of each workshop. The evaluation survey had 12 items on the relevancy and usefulness of the workshop’s content regarding the medical specialty exam and level of satisfaction with the workshop. It was possible to include open-ended responses for the following questions:

- Do you have any suggestions for improving the forms and/or materials?
- For which aspects would you like more information and/or support?
- What was the most important thing you learned from this workshop?

Analysis of the open-ended responses included sorting and categorizing as the first step. Then, four different raters independently assigned the responses to the pre-defined categories.

## Results

Of the 449 examiners registered with the Regional Medical Association for South Baden only around 250 are active, and only ten of the 33 head examiners. Of these 260 active examiners and head examiners, 93 (36%) have taken part in the training program. Of these, 42 participants (52.5%) indicated they had more than five years of experience as an examiner, ten participants (12.5%) indicated three to five years, and 12 (15%) between one and two years. Eleven participants (13.7%) had not yet administered an exam, and five (6.3%) gave no indication of experience.

The specific medical specialties of the examiners who participated in the six training programs are presented in table 2 (Tab. 2). Mostly, the examiners are specialized in internal medicine, surgery, general practice, gynecology, and emergency medicine. 

**Figure 1:**
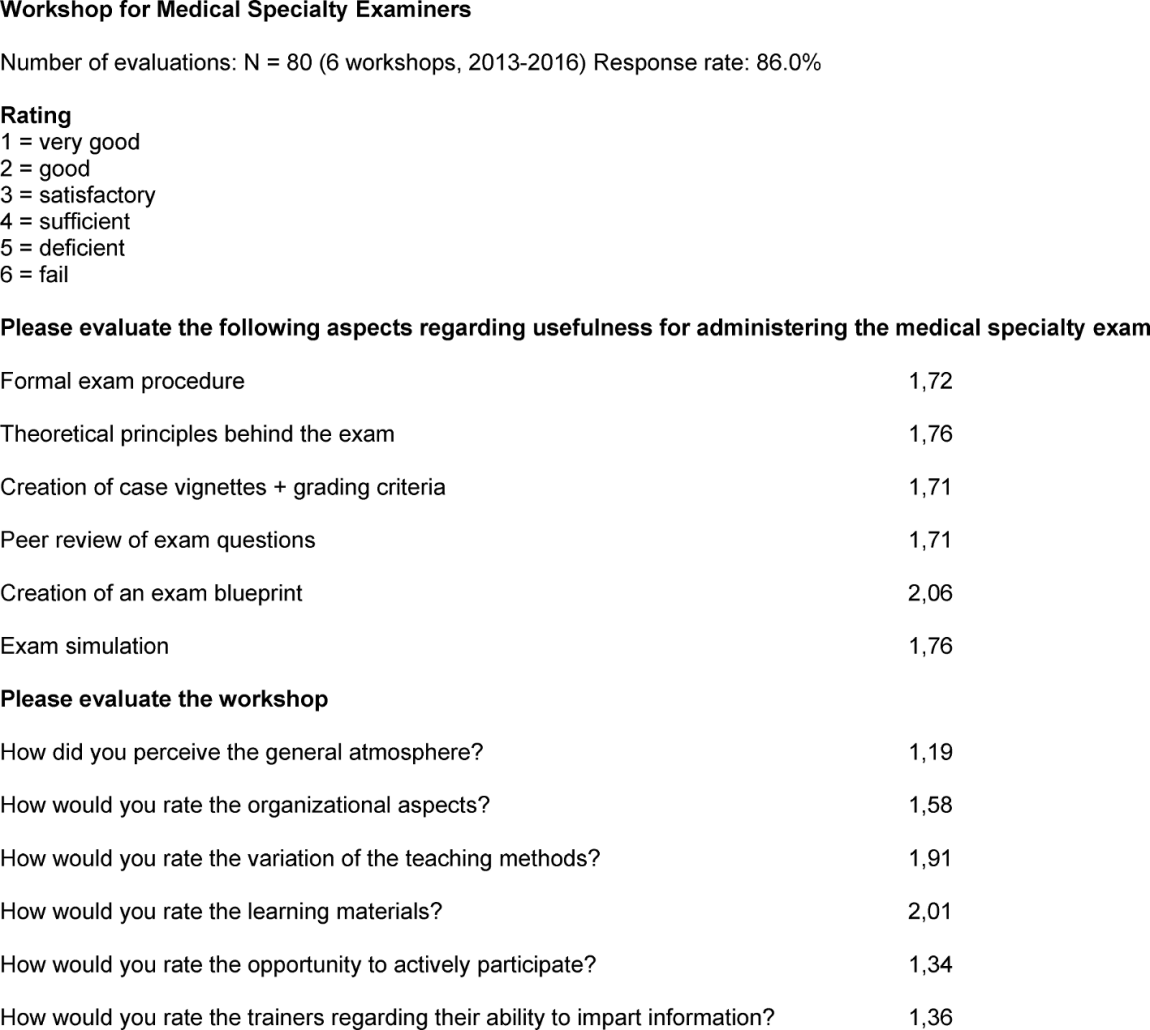
Evaluation of the examiner workshops, 2013-2016 (N=80)

The evaluations administered after each training session (response rate: 86.0%) showed a high level of acceptance for the training strategy, primarily the opportunity to actively participate during the workshop and the positive atmosphere among medical colleagues. In addition to the structured presentation of information and the theoretical principles underlying the need for a structured oral exam, the participants found the generation of case vignettes, the internal review of the exam questions by medical colleagues and the simulated exam especially useful (see figure 1 (Fig. 1)).

The most meaningful result to arise from analysis of the open-ended responses was seen in answers to the question about the most important insight from the workshop. Participants primarily found proposals to standardize and formally structure exams to be meaningful. Likewise, exchange and coordination between examiners for a specific specialty was of great importance. A verifiable and comparable structuring of the medical specialty exams, standardization of grading, prior coordination among examiners, and the creation of an internal catalogue of case vignettes for each specialty were explicitly mentioned. In addition, the examiners and head examiners had a clearer understanding of the legal and formal aspects of the medical specialty exam. Many experienced examiners felt affirmation regarding how they had previously handled exams but were still able to take away additional suggestions from the workshops for future exams.

**Figure 2:**
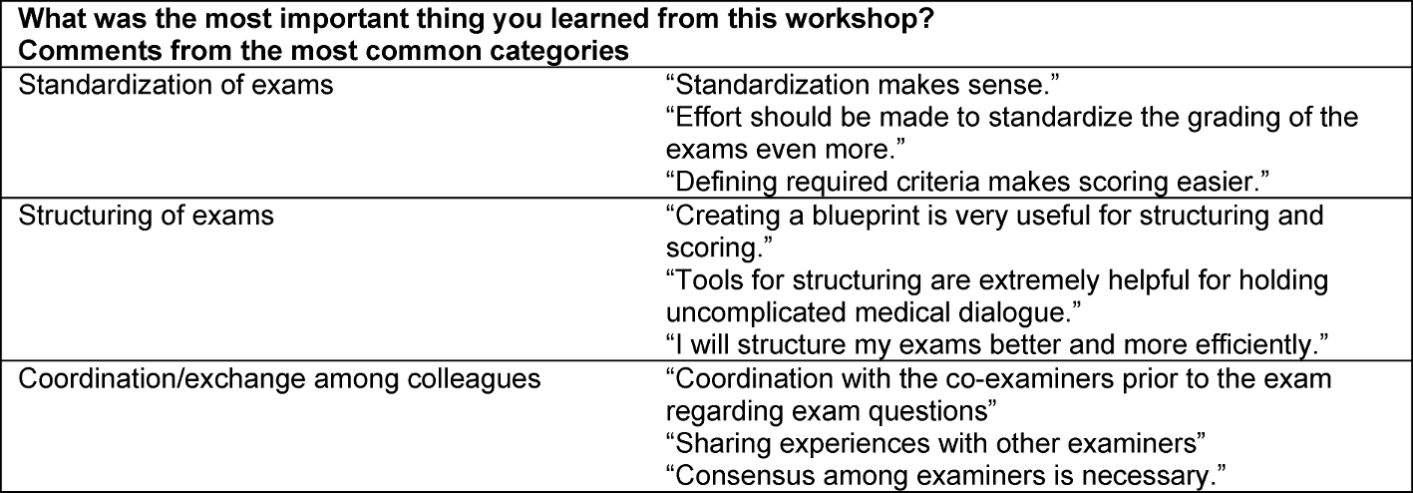
Selected responses to questions about the most important personal observations from the workshop.

Several open-ended responses on the most important insights from the workshops are listed in figure 2 (Fig. 2) as examples from the three most frequently named topics (standardization, structuring and coordination, and professional exchange with other examiners for the same specialty):

## Discussion

The evaluations at the end of the examiner workshop showed that the examiners see a need to develop shared minimum standards and a uniform level of requirements for each medical specialty and medical specialty exam and, above all, to give examiners new tools that enable improvements in the objectivity, reliability, and thus validity of the oral assessments [1], [2], [4]. The need for good structuring and standardization appeared particularly important to the examiners in terms of the oral exam. This can primarily be seen in the subjective assessments of the usefulness of the case vignettes, their peer review and the many comments on the most important observations made during the workshops (see figure 2 (Fig. 2)).

Öchsner et. al come to a similar result in a study they conducted in Ulm, Germany, on the effects and sustainability of a training workshop aimed at the M2 exam [10]. In that study, 86% of the workshop attendees indicated that they strived to apply the concept of the “structured oral examination.”

The simulated exam as a practical application of the workshop content, and thus a central element of the medical specialty examiner workshop, was viewed by the examiners as extremely useful for the administration of the medical specialty exam.

A second evaluation of the medical specialty exams with a survey of the examiners, head examiners, and examinees is planned with a focus on answering the following questions:

- Were changes initiated in how the examiner conducted exams as a result of the workshop, and if so, what changes?

Possible criteria for this would include the creation of a shared, subject-specific blueprint and definition of the grading criteria for passing and failing scores.

- Are there differences between examiners who have undergone training and those who have not when administering medical specialty exams?

Possible criteria for this would be whether or not written case vignettes including previously defined grading criteria had been created, if these were used during the exam, and if the formal criteria for a good case vignette had been fulfilled.

The <3% failure rate on the medical specialty exam prior to implementation of the examiner workshop has shown no substantial change in the past three years, so that it must be investigated if this criteria can be drawn upon as a quality marker for a changed testing culture.

If the published failure rates for exams leading to additional qualifications in medical disciplines and specialties given by the different medical associations across Germany are considered, then it is possible to see that these rates vary significantly. Not all of the state medical associations provide these numbers annually or in a transparent manner, and the published failure rates range widely, for instance, from 3.5% in Hamburg [11] to 6.4% in Westfalen-Lippe [12] for all types of specialty exams and from 2.5% in Hamburg [11] to 7.2% in Westfalen-Lippe [12] for exams leading only to an additional occupational designation in a medical specialty.

### Analysis of strengths and weaknesses

The strengths of this project lie in the fact that the training program for medical specialty examiners is the first of its kind in Germany, and thus acts as a role model. For example, the representatives of the Regional Medical Association for South Baden have not only contemplated qualitative improvements to the medical specialty exams earlier than other medical associations, but have also decided to offer and implement appropriate training for examiners and head examiners. In addition, a successful workshop design was developed, one that has been very positively received by the participants.

One of the challenges faced by this project was that the workshop participants came from a wide variety of medical specialties so that it was not always possible to bring at least four examiners from a single specialty (see table 2 (Tab. 2)) together for professional exchange on assessment objectives or exam content or to carry out a simulated exam as part of the workshop. During the first training sessions, this was only a small problem – it had been possible to invite a sufficient number of representatives from three to four medical specialties. In recent training sessions, however, it has become more frequent that only a few representatives have participated in the workshop. In these cases it was necessary for the trainers to closely mentor the representatives during the review phase. During the simulation, these representatives are only able to serve in an observational capacity.

One further limitation is found in that up until now no outcome could be assessed in respect to the training. This is being considered in follow-up investigations.

## Conclusions

Training programs for examiners administering medical specialty exams and other assessments leading to additional post-licensure medical qualifications appear to be both valuable and necessary despite, or perhaps precisely because of, the low failure rates. Since failure rates for these exams vary widely across Germany, aiming for examiner qualification would be worth considering, not just in South Baden, but also in other regions, as this is already standard in other European countries. For improvement of quality across the board, it seems important to develop and implement a uniform concept for all of Germany.

Regardless, the establishment of other assessment procedures for post-licensure medical specialization should also be considered in Germany. Relying only on knowledge-based tests is not sufficient to assess and document the competencies required of medical specialists. It is much more critical to assess how medical expertise is applied in the practical setting [4]. Examples from other European countries point to a better measurement of competency by using longitudinal formats [6]. For this reason, it would make sense to consider a new assessment format for the medical specialty exams in Germany and implementing a combination of multiple longitudinal assessments as well as a final exam. Similar considerations have been voiced in the position paper published in 2013 by the GMA committee on post-graduate education [13]. In addition to this, a 90-minute assessment at the end of the specialist training, such is already done in Thuringia [7], could be considered.

These new assessment formats could replace the current 30-60 minute-long oral examination in front of two specialist physicians and a head examiner. However, any changes to the assessment format will be accompanied by an increase in costs, as is expressly mentioned by Flum et. al in 2015 [6].

## Note

Dr. med. Irmgard Streitlein-Böhme, Dr. med. Götz Fabry and Dr. med. Klaus Böhme received financial compensation from the Regional Medical Association for South Baden to develop and conduct the examiner workshops.

## Competing interests

The authors declare that they have no competing interests. 

## Figures and Tables

**Table 1:**
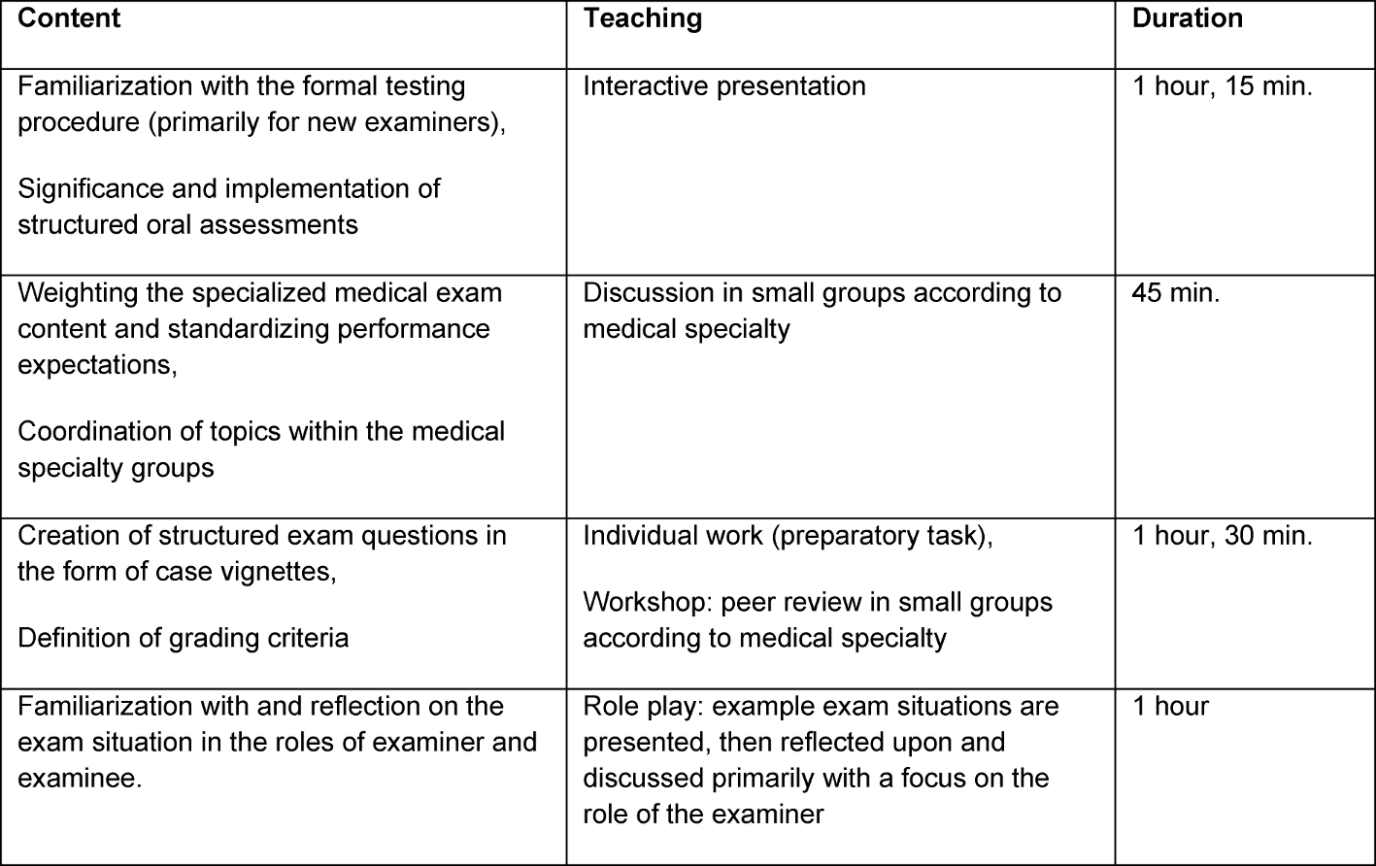
Organization of the workshops in terms of content and teaching

**Table 2:**
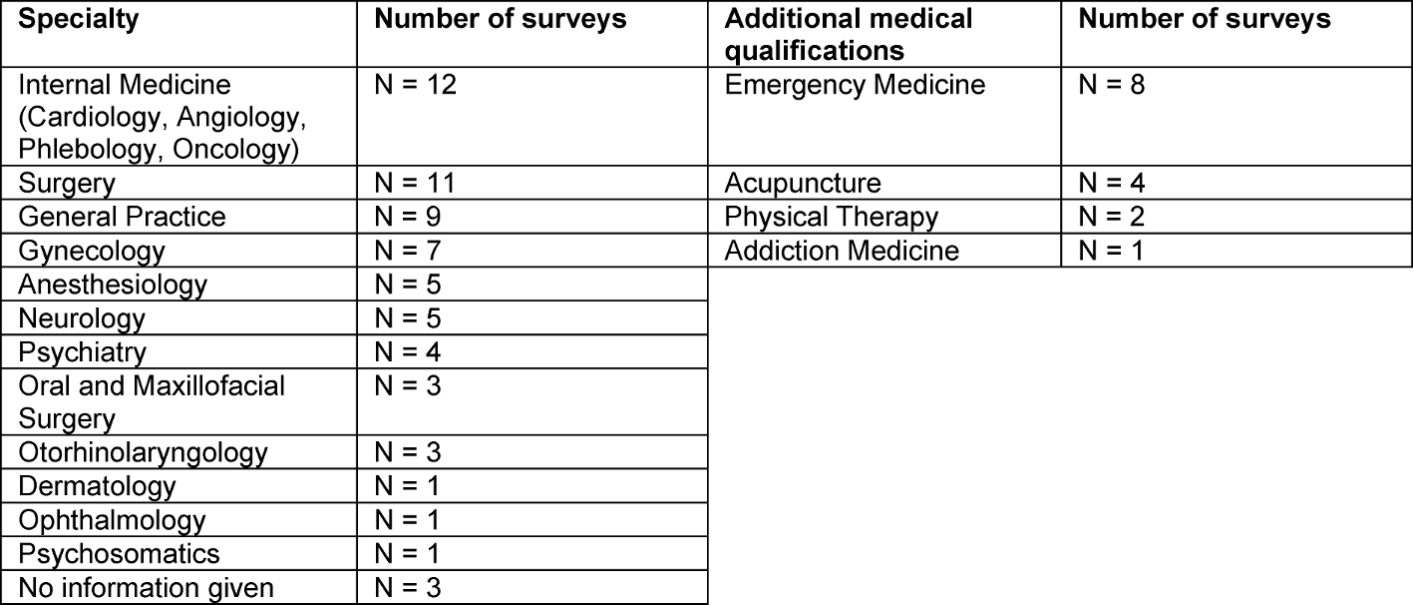
Distribution of subjects in the evaluation surveys analyzed (N=80)
